# Leukocytes within Autologous Blood Concentrates Have No Impact on the Growth and Proliferation of Human Primary Osteoblasts: An In Vitro Study

**DOI:** 10.3390/ijms25084542

**Published:** 2024-04-21

**Authors:** Carlos Fernando Mourão, Eva Dohle, Büşra Bayrak, Anne Winter, Robert Sader, Shahram Ghanaati

**Affiliations:** 1 Department of Periodontology, Tufts University School of Dental Medicine, Boston, MA 02111, USA; carlos.mourao@tufts.edu; 2 FORM—Frankfurt Orofacial Regenerative Medicine, Department for Oral, Cranio-Maxillofacial and Facial Plastic Surgery, Medical Center of the Johann Wolfgang Goethe University, 60590 Frankfurt, Germany; bayrak.busra@icloud.com (B.B.); anneewi10@gmail.com (A.W.); sader@em.uni-frankfurt.de (R.S.)

**Keywords:** blood concentrates, platelet-rich fibrin, PRF, L-PRF, plasma rich in growth factors, osteoblasts, bone regeneration

## Abstract

Platelet-rich fibrin (PRF) is a widely used autologous blood concentrate in regenerative medicine. This study aimed to characterize the cellular composition and distribution of different PRF matrices generated by high (710 g) and low (44 g) relative centrifugal forces (RCFs) and to analyze their bioactivity on human primary osteoblasts (pOBs). PRF was separated into upper layer (UL) and buffy coat (BC) fractions, and their cellular contents were assessed using histological and immunohistochemical staining. The release of platelet-derived growth factor (PDGF) and transforming growth factor (TGF-β) was quantified using an ELISA. Indirect PRF treatment on pOBs was performed to evaluate cell viability and morphology. A histological analysis revealed higher quantities of leukocytes and platelets in the low-RCF PRF. TGF-β release was significantly higher in the low-RCF PRF compared to the high-RCF PRF. All PRF fractions promoted pOB proliferation regardless of the centrifugation protocol used. The low-RCF PRF showed higher TGF-β levels than the high-RCF PRF. These findings contribute to understanding the cellular mechanisms of PRF and provide insights into optimizing PRF protocols for bone regeneration, advancing regenerative medicine, and improving patient outcomes.

## 1. Introduction

Over the last 20 years, regenerative medicine has undergone a remarkable transformation with the introduction of autologous blood concentrates in the medical and dental fields [1,2,3]. These concentrates have evolved from first-generation platelet-rich plasma (PRP) [4,5] plasma rich in growth factors (PRGF) to second-generation platelet-rich fibrin (PRF) [6]. In the early 2000s, PRF was introduced by Choukroun et al. [6], and it revolutionized the field by simplifying the preparation process. It eliminated the need for anticoagulants and external additives and harnessed the body’s natural coagulation pathway. This approach resulted in a fibrin matrix that not only served as a scaffold for immune cells but also facilitated the sustained release of cytokines and growth factors which could promote long-term healing and tissue regeneration [7,8].

The shift from PRP to PRF was a significant change in how we use the body’s natural healing mechanisms. PRF is known for its autologous nature, which means that it uses the patient’s own blood to continuously release bioactive molecules [9,10,11]. This unique ability makes PRF an essential tool in clinical practice, allowing for more efficient and natural healing to occur in bone and soft tissue regeneration procedures. Decades of research and technological advancements have led to this evolution, which reflects a broader trend toward optimizing biologically based healing strategies [12]. Advances in blood concentrates in regenerative medicine have created new opportunities for patient care and therapeutic interventions (i.e., the treatment of diabetic foot ulcers) [12,13].

Blood concentrates were developed and applied, seeking the help of leukocytes and platelets, which play crucial roles in tissue healing. Leukocytes are the body’s primary defense against infections, while platelets are essential for blood clotting and growth factor release [14,15]. Together, they work synergistically in blood concentrates to significantly improve tissue healing. This is achieved by providing a scaffold for new tissue growth and the enrichment of essential growth factors and cytokines. The PRF-mediated biological improvement of the environment contributes directly to better healing outcomes, shorter recovery times, and a reduced risk of complications (e.g., infections) [16,17].

The original idea for producing PRF involved utilizing a relative centrifugal force (RCF) of approximately 710 g for 8 min in a vertical centrifuge. This process was introduced as a high-RCF method for PRF production [17]. By introducing low-speed centrifugation (low-RCF) techniques to refine PRF protocols and enhance their regenerative potential, the concentrations of key cellular components, particularly neutrophils and platelets within PRF matrices, could be increased [17]. Therefore, a protocol was created to amplify the synergistic effects that are essential for boosting PRF’s biological activity and therapeutic efficacy. This approach focuses on optimizing the concentration of immune cells in PRF, which has been demonstrated to be a sophisticated strategy for harnessing the body’s natural healing mechanisms for better clinical results in tissue engineering and regenerative medicine [17].

Thus, blood concentrates have become an important component of regenerative medicine, particularly in the context of bone and soft tissue healing, by accelerating the healing process and enhancing the quality of regenerated tissue. This biological scaffolding has been demonstrated to be effective in supporting tissue engineering, making it a versatile tool in various clinical applications [18]. However, there are many other different protocols available, each claiming superiority in certain aspects of healing. This makes it challenging for clinicians to choose the best protocol for their patients. To select the optimal protocol, practitioners must carefully consider the specific clinical scenario, the patient’s unique needs, and the evidence supporting the efficacy of each option. This decision-making process is essential in harnessing the full therapeutic potential of blood concentrates to achieve the best possible outcomes in tissue regeneration [19].

The main goal of the present study is to characterize the cellular composition and cellular distribution of different portions of a blood concentrate generated using two different relative centrifugal forces with the same spinning time, separating their layers (the upper layer (UL) and buffy coat (BC)) and analyzing their bioactivity regarding the proliferation of human primary osteoblasts. Within this, the research seeks to investigate the unique contributions of each layer of these two PRF protocols by unraveling their composition and functional heterogeneity. By doing so, this research will provide insights into the optimal utilization of each portion, guiding future clinical applications toward improved efficacy in bone and soft tissue healing.

This investigation aims to elucidate the cellular (leukocyte) composition through which blood concentrates exert their regenerative effects and to refine the selection criteria for their protocols. This will align them more closely with the specific requirements of targeted therapeutic interventions. Ultimately, this study seeks to contribute to the ongoing endeavor of advancing regenerative medicine and improving patient outcomes through the evidence-based optimization of blood concentrate therapies.

## 2. Results

### 2.1. Cellular Characterization of Different Fractions of High- and Low-RCF PRF

**Figure 1:**
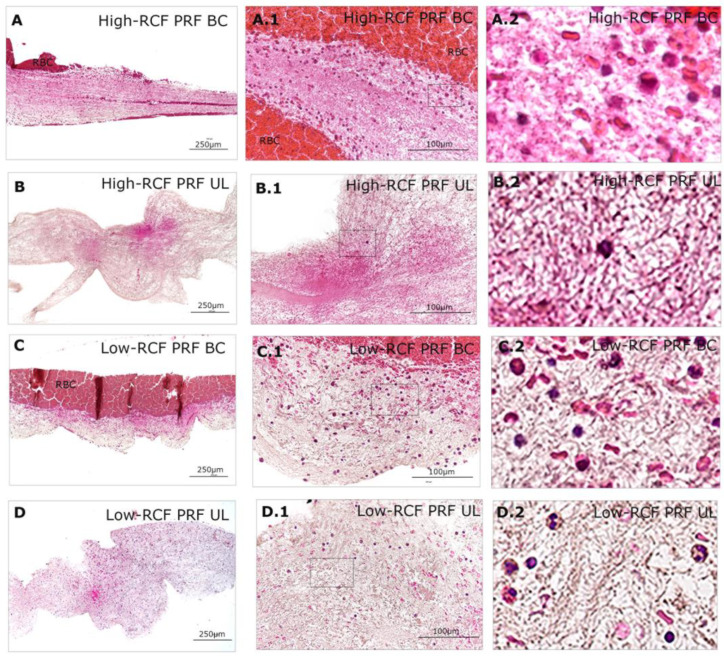
Histological overview staining of different fractions (upper layer (UL) and buffy coat layer (BC)) of high- and low-RCF PRF for H&E images at different magnifications. Scale bars: (**A**–**D**) = 250 µm and (**A.1**–**D.1**) = 100 µm. RBC = red blood cell. (**A.2**–**D.2**) = an enlarged representative area of the rectangles in (**A.1**–**D.1**), respectively.

In order to characterize different PRF fractions in terms of their cellular content and composition, samples were histologically and immunohistologically stained for a panel of different markers: H&E, neutrophil elastase, and interferon gamma, as well as CD61. In general, the buffy coat parts, which are close to the red blood cells, contain a number of immune cells like leukocytes, especially neutrophils, which are documented by H&E staining and independent of the RCF used for the generation of the PRF. Lower magnifications of H&E stains (Figure 1) provide a brief overview of the composition/arrangement of the whole fractions (UL and BC) of the different RCF PRF clots. Figure 1A,C represent the denser BC fraction close to the red blood cells (RBCs) with, generally, a higher number of cells, whereas the upper layer (UL) of the different RCF PRFs appears more loosely arranged, and no red blood cells can be found (Figure 1B,D). When compared to the upper layer (UL) fractions of both RCF PRF samples, generally, more cells can be documented when generating the PRF with a low RCF (Figure 1D) compared to the high-RCF PRF, in which nearly no cells can be documented within the UL fibrin (B).

**Figure 2:**
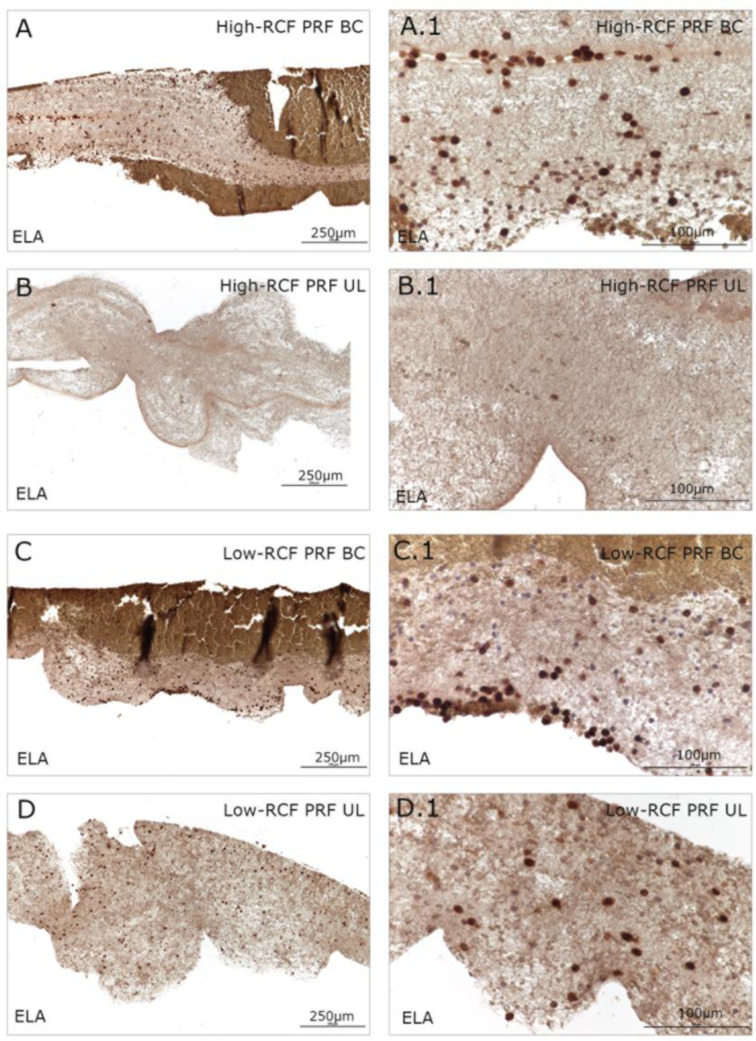
Immunohistological staining of different fractions (upper layer (UL) and buffy coat layer (BC)) of high- and low-RCF PRF for neutrophil elastase (ELA) to visualize the neutrophils at different magnifications. Scale bars: (**A**–**D**) = 250 µm and (**A.1**–**D.1**) = 100 µm.

**Figure 3:**
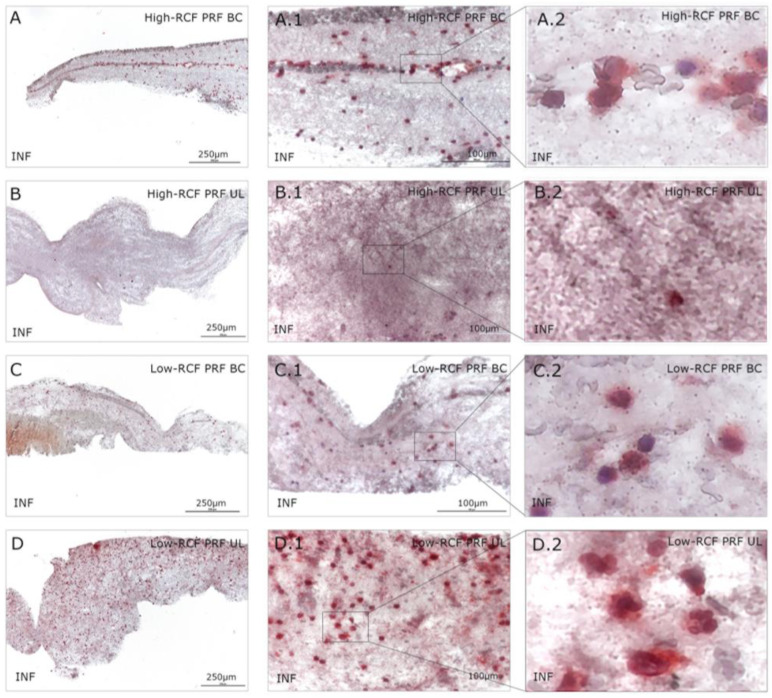
Immunohistological staining of different fractions (upper layer (UL) and buffy coat layer (BC)) of high- and low-RCF PRF for interferon gamma (INF) to visualize the proinflammatory neutrophils in different magnifications. Scale bars: (**A**–**D**) = 250 µm and (**A.1**–**D.1**) = 100 µm. (**A.2**–**D.2**) = an enlarged representative area of the rectangles in (**A.1**–**D.1**), respectively.

**Figure 4:**
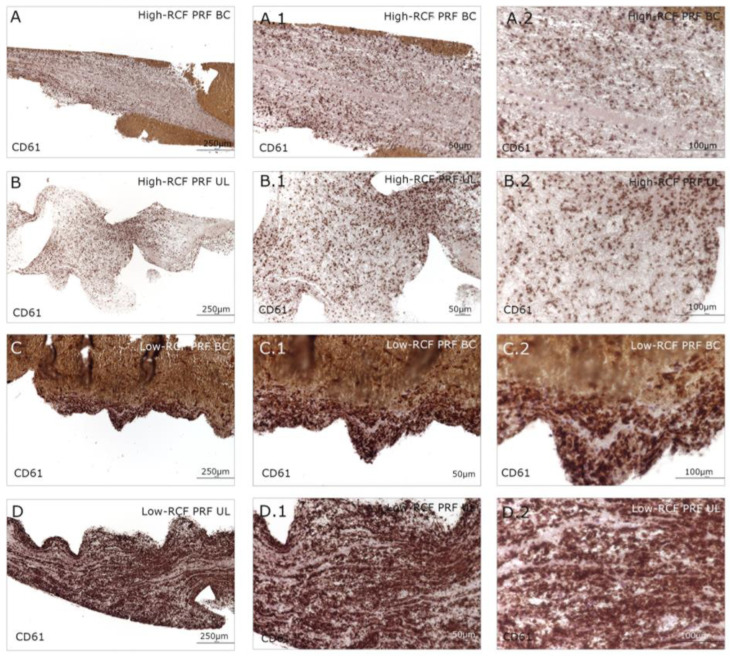
Immunohistological staining of different fractions (upper layer (UL) and buffy coat layer (BC)) of high- and low-RCF PRF for CD61 to visualize the platelets in different magnifications. Scale bars: (**A**–**D**) = 250 µm and (**A.1**–**D.1**) = 50 µm and (**A.2**–**D.2**) = 100 µm.

Immunohistologically stained samples for neutrophil elastase (Figure 2) and interferon (Figure 3) as markers of neutrophils revealed no visible difference in total cell amount related to the RCF in the buffy coat parts of both RCF PRF samples. Significant more leukocytes/neutrophils can be visualized in the upper fraction of the low-RCF PRF sample compared to the UL of the high-RCF PRF; in general, nearly no positive stained cells can be detected. In order to estimate the content and distribution of platelets within the different fractions of high- and low-RCF PRF, immunohistological staining for CD61 was performed. A high amount of CD61-positive cells can be visualized in both parts of the low-RCF PRF (BC and UL) (Figure 4C,D). In contrast, the high-RCF PRF contains fewer CD61-positive cells, and their numbers are roughly comparable in the BC and UL fractions (Figure 4A,B). 

### 2.2. Determination of Growth Factor Release of Different PRF Fractions/Layers

**Figure 5:**
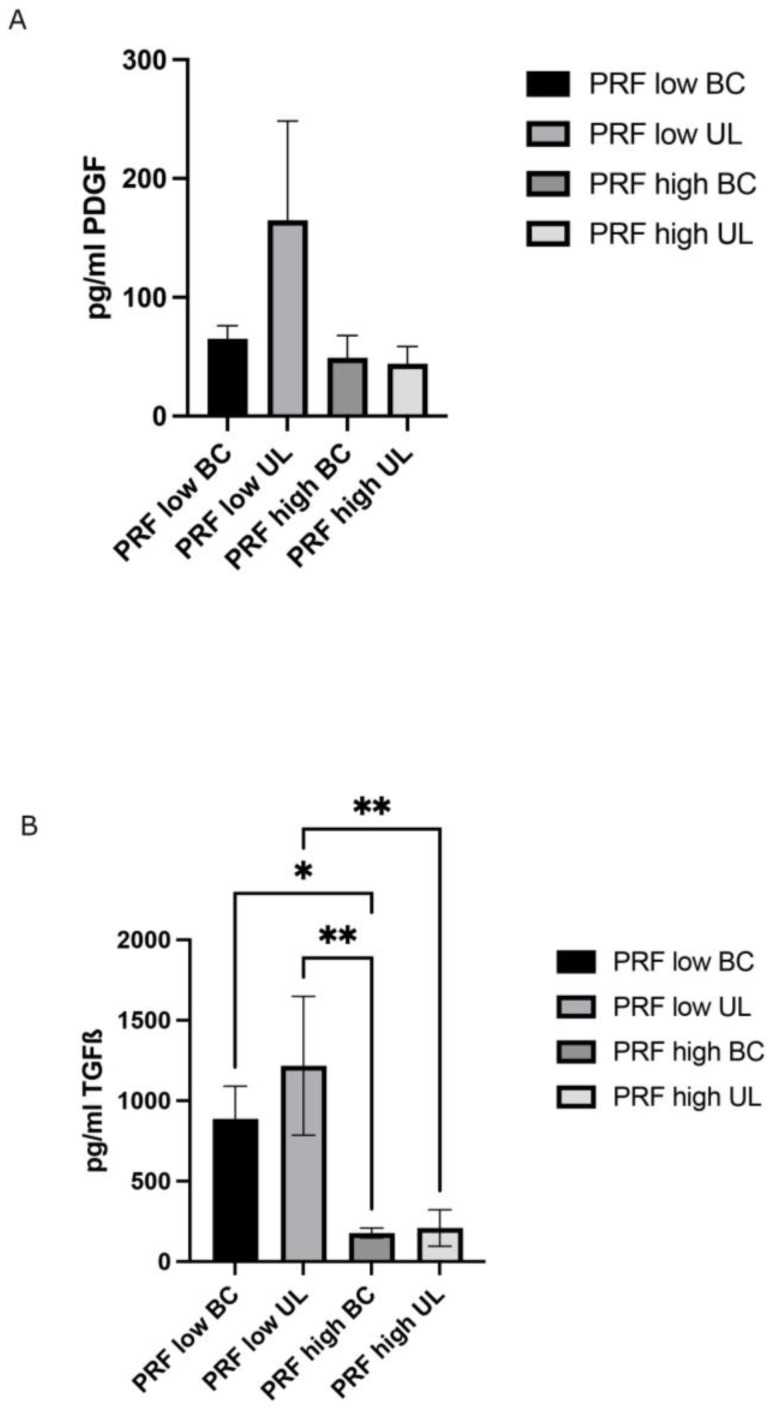
Determination of growth factor release of different fractions (upper layer (UL) and buffy coat layer (BC)) of high- and low-RCF PRF after 2 days of cultivation. (**A**) PDGF and (**B**) TGF-β. * *p* < 0.05; ** *p* < 0.01.

To further characterize the different fractions of both high- and low-RCF PRF, the release of PDGF and TGF-β was determined in cell culture supernatants using an ELISA (Figure 5A,B). Although the concentration of the PDGF slightly differed between the different experimental groups, no statistically significant difference in PDGF release could be assessed (Figure 5A). The PDGF concentration in the supernatants of the analyzed PRF fractions ranged from ~70 to ~160 pg/mL. The TGF-β release was significantly higher in the supernatants of the low-RCF PRF UL and low-RCF PRF BC (~800 and ~1200 pg/mL) than in the UL and BC fractions of the PRF generated via high RCF (~170 and ~200 pg/mL) (Figure 5B). Furthermore, no significant difference in TGF-β release was observed between the appropriate UL and BC fractions of either RCF PRF.

### 2.3. Comparative Bioactivity Analysis of Different PRF Fractions on pOBs

**Figure 6:**
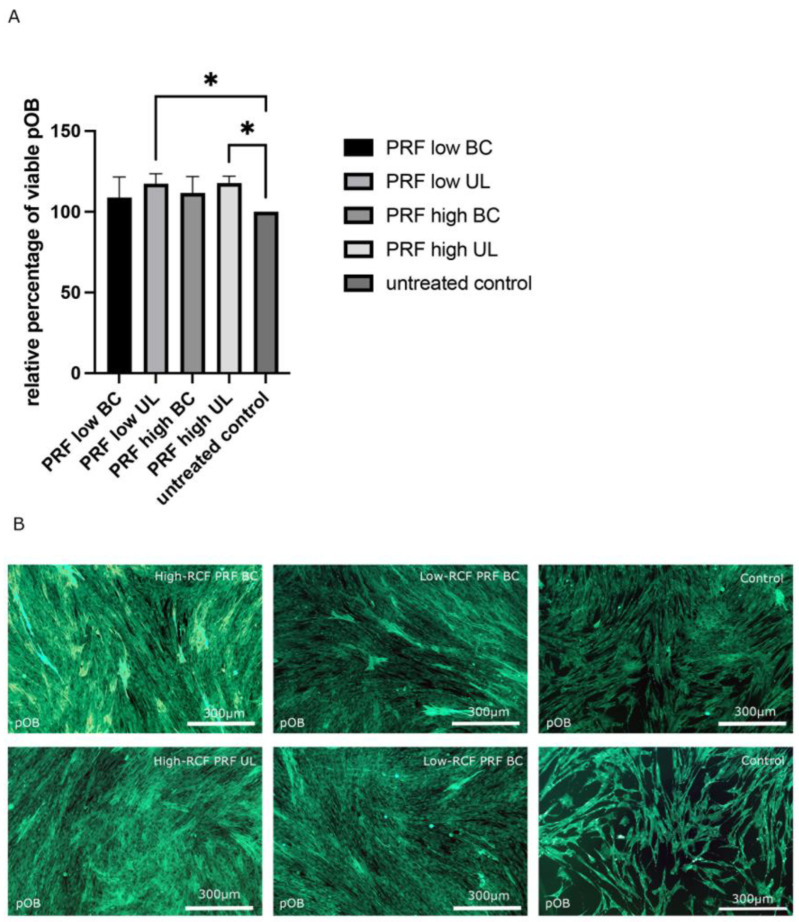
(**A**) Cell viability of pOBs in response to indirect treatment with different fractions (UL and BC) of high- and low-RCF PRF samples for two days compared to untreated control, * *p* < 0.05. (**B**) Cell type-specific immunofluorescence for smooth muscle actin to visualize the pOBs to untreated control. Scale bars B: 300 µm.

Monoculture pOBs were indirectly treated with different fractions of PRF (high- and low RCF (UL and BC)) for two days to investigate the effects of the different PRF fractions on the morphology and proliferation of pOBs. In general, cell viability increased in all experimental PRF groups compared to the untreated control pOBs (Figure 6A). Nevertheless, statistically significantly higher cell viability could be assessed when the pOBs were treated with the upper layer (UL) of both high and low PRF treatments compared to the control. In addition, the pOBs were stained with smooth muscle actin antibody (SMA) via immunofluorescence staining (Figure 6B). 

Confirming the results regarding cell viability, a higher cell number was observed in pOBs treated with PRF, independent of the PRF fraction used for the treatment, compared to pOBs without PRF treatment, in which significantly fewer pOBs could be visualized. Primary osteoblasts treated with the BC and UL fractions of low- and high-RCF PRF formed a confluent cell layer covering the entire bottom of the cell culture well. In pOBs without PRF treatment, the cell layer was semiconfluent, with irregularly distributed cellular gaps.

## 3. Discussion

Blood concentrates, such as PRP and PRF, have become increasingly popular in dentistry and regenerative medicine for improving wound healing and regeneration processes [1,12,19,20]. PRP, a blood concentrate that has been studied extensively for tissue regeneration, has shown conflicting results regarding its effect on bone neoformation [21,22]. Some studies suggest a positive effect, while others indicate no synergistic effect on bone formation [20]. It is worth noting that the results of the present study, which utilized PRF, are consistent with those of a prior study that used PRP with a greater concentration of leukocytes (L-PRP) [23]. In that study, an increase in the activation of the nuclear factor κB (NF-κB) pathway was observed, promoted by an increased concentration of pro-inflammatory cytokines, which had a negative impact on bone regeneration when compared to regular PRP, which had a lower concentration of leukocytes [23].

PRF contains important immune cells and growth factors with regenerative properties [14,15,17,18]. In this study, PRF was separated into two different layers (UL and BC) to evaluate their effectiveness and determine if one layer had superior activity on primary osteoblasts in vitro compared to the other. The study found that all PRF layers from the different protocols applied were more effective in promoting pOB proliferation than the control. This supports previous research demonstrating that PRF can enhance osteoblast viability and proliferation regardless of the specific protocol used, indicating that different layers of the whole PRF matrix can promote osteoblast proliferation [24,25]. One of the most important outcomes of this study was the fact that the presence of any concentration of cells, or even the absence of leukocytes, as observed in the high-RCF UL, stimulated osteoblasts to proliferate when treated with all layers of the PRF used in the experiment. This is an interesting finding because according to present research, any PRF layer will be able to help in cell proliferation. However, even more viable cells could be found in the plasma-treated groups’ ULs compared to the untreated control and the BC-treated pOBs. 

A histological analysis revealed a similar quantity of leukocytes in the low-RCF BC, and UL and the high-RCF BC. The researchers used neutrophil elastase (ELA) and interferon gamma (INF) to identify neutrophils in the PRF layers and found a higher quantity of neutrophils in all groups except the UL of the high-RCF PRF. INF is typically a marker of the inflammatory process [26]; however, in this study, the authors used it to identify pro-inflammatory neutrophils. While neutrophils are powerful pro-inflammatory cells in the acute phase, they also play a role in resolving inflammation and supporting key repair processes, such as revascularization and the recruitment of pro-resolving monocytes/macrophages, in later stages [27]. The timely recruitment and removal of neutrophils seem to be crucial for optimal tissue regeneration and healing after injury or infection [28]. The presence of these cells in PRF may be advantageous for certain applications, such as serving as an adjuvant in treating infections, which could account for the antimicrobial activity of PRF [16]. In addition, the growth factors present in PRF, such as TGF-β, support this effect by positively stimulating osteoblasts for collagen synthesis [29]. The higher concentrations of platelets and leukocytes in the low-RCF PRF group, as demonstrated by immunohistochemistry, may contribute to enhanced tissue regeneration due to the increased release of the growth factors and cytokines involved in these processes. It was not possible to confirm this hypothesis when separating the PRF layers in the present study in the presence of pOBs.

The growth factors assessed in this study showed no significant differences in PDGF levels between groups. However, TGF-β levels were notably higher in the low-RCF PRF group than in the high-RCF PRF group. Specifically, TGF-β was significantly elevated in the low-RCF BC compared to the high-RCF BC (*p* < 0.05) and in the low-RCF UL compared to both the high-RCF BC (*p* < 0.01) and the high-RCF UL (*p* < 0.01). These findings suggest that the low-RCF PRF protocol may enhance the release of TGF-β, a key growth factor in bone and soft tissue regeneration. TGF-β is known to stimulate osteoblast proliferation, differentiation, and matrix synthesis, as well as inhibit osteoclast formation and activity. TGF-β also plays a crucial role in angiogenesis by promoting endothelial cell proliferation, migration, and tube formation [30]. Collectively, these effects contribute to enhanced bone regeneration. The higher TGF-β levels in the low-RCF PRF groups are consistent with previous studies that demonstrated increased growth factor release in PRF prepared using lower centrifugation speeds. These findings underscore the importance of optimizing PRF protocols to maximize the release of key growth factors involved in tissue regeneration. On the other hand, despite the higher liberation of the TGF-β growth factor in the low-RCF BC group, there was no demonstrated increase in pOB proliferation when treated using this group.

For PDGF growth factor release, no significant differences between the groups could be assessed. However, immunohistochemistry staining with a CD61 antibody showed a significantly higher quantity of platelets in the low-RCF PRF group, especially in the UL, compared to the high-RCF PRF group. This finding is consistent with previous studies that demonstrated that low-speed centrifugation results in higher concentrations of platelets and leukocytes. This was observed by combining the results of a histological evaluation of PRF matrices. Platelets play a pivotal role in PRF-mediated tissue regeneration by releasing an oversupply of growth factors and cytokines, such as PDGF, TGF-β, VEGF, and EGF, which collectively stimulate cell proliferation, migration, differentiation, and angiogenesis [13,18,19,25,31]. The higher platelet content in the low-RCF PRF, particularly in the UL, suggests an enhanced regenerative potential compared to the high-RCF PRF. These findings have been demonstrated in several previous studies. However, in the present study, even with a high quantity of platelets, cell proliferation remained similar when using high- or low-RCF PRF. Moreover, various studies have demonstrated that the presence of leukocytes in PRF also contributes to its regenerative properties through the release of additional growth factors and the modulation of the inflammatory response [31,32,33,34]. Future studies should explore the use of different protocols for specific clinical applications. 

This study is the first to separate the layers of a blood concentrate in order to assess the relationship between cellularity and the proliferation of pOBs in each portion (UL and BC) without the inclusion of anticoagulants. There is a prior comparison between the first generation of blood concentrates (PRGF) and the second generation (PRF). In that study, the authors emphasized that the presence of leukocytes significantly increases the secretion of proinflammatory cytokines. The researchers hypothesized that this could negatively impact the synthesis of type I collagen and alkaline phosphatase by osteoblasts, impacting new bone formation [29]. However, the present research did not evaluate these proteins because our target was to evaluate pOB proliferation with or without leukocytes in the blood concentrate. 

In addition, it is crucial to emphasize the groundbreaking results of the present study because some previous studies highlighted the importance of leukocytes in bone regeneration and their role in releasing cytokines and growth factors for osteoblast proliferation during new bone formation. In addition, many authors have used different names for blood concentrates, regardless of how the concentrate is generated (e.g., PRP, L-PRF, H-PRF, and C-PRF) [35,36,37,38,39]. It is confusing to some readers, especially for clinicians who work with blood concentrates in their daily practice. Thus, the results of this study demonstrate that regardless of the number of leukocytes and independently of the protocol used, pOB proliferation is possible. This highlights that there are other factors related to blood concentrates beyond the presence of leukocytes. In future studies, the authors aim to investigate the role of platelets and the fibrin matrix in blood concentrates in bone regeneration.

The clinical relevance of the present study addresses a common question among clinicians: Is it possible to improve osteoblast proliferation using different PRF layers and protocols, and which PRF protocol is the most effective for enhancing osteoblast differentiation and/or bone formation? Based on the findings of this study, the answer to both questions is that any PRF protocol might be useful. The results demonstrated that all PRF layers in the present study, including UL and BC, exhibited similar properties in promoting the proliferation of pOBs in vitro. This suggests that, clinically, either UL or BC can be used to stimulate osteoblast proliferation and, consequently, bone formation.

One of the main concerns regarding the use of blood concentrates in bone regeneration is the need for blood cells to promote the increase in cytokines and growth factors which subsequently leads to new bone formation [40,41,42,43]. While previous studies have shown that blood concentrates can improve osteoblast activity, this groundbreaking discovery is that any layer of PRF, regardless of the centrifugation protocol used, can achieve this effect.

To facilitate clinicians’ understanding, the present study indicates that it is possible to produce “sticky bone” using any PRF protocol regardless of whether it involves high or low RCF. This sticky bone will aid clinicians in handling biomaterial during surgical procedures [40,42]. However, future studies should investigate the effects of high- and low-RCF protocols with various bone substitutes on new bone formation. Focusing on biodegradation capacity, which can be promoted or impaired by the presence of neutrophil granulocytes, these findings provide valuable insights into the versatility and effectiveness of different PRF protocols for promoting osteoblast proliferation and bone regeneration.

The present study had some limitations that should be acknowledged. First, this study was conducted in vitro using primary human osteoblasts, which may not fully represent the complex in vivo environment of bone regeneration. In vitro models, which are valuable for understanding cellular mechanisms, do not account for interactions between different cell types, the presence of the extracellular matrix, or the influence of systemic factors. In addition, this study focused on osteoblast proliferation, which is only one aspect of bone regeneration. Other crucial processes, such as osteoblast differentiation, mineralization, and the formation of a functional bone matrix, were not evaluated. Furthermore, this study did not assess the potential impact of different PRF protocols on osteoclast activity, which plays a crucial role in bone remodeling. Another limitation is that this study did not explore the potential synergistic effects of combining PRF with other bone substitutes (sticky bone) commonly used in bone regeneration. Finally, this study did not include an in vivo component, which would have provided valuable insights into the clinical relevance of the findings. Future studies should address these limitations by incorporating more complex in vitro models, evaluating additional aspects of bone regeneration, exploring a wider range of PRF protocols and combinations, and validating their findings in well-designed experimental studies and clinical trials.

## 4. Materials and Methods

### 4.1. Preparation and Separation of the Blood Components

**Figure 7:**
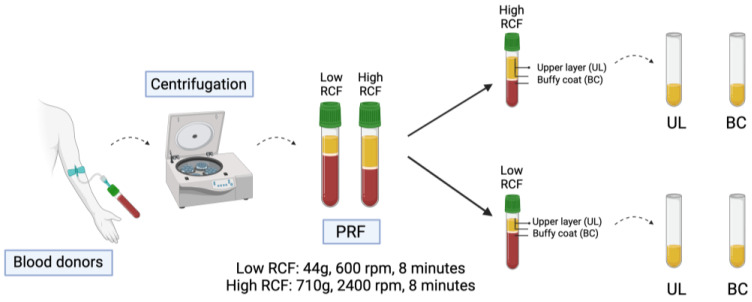
Flow diagram of preparation and separation of PRF components.

Whole blood was collected from 3 donors of all genders who gave informed consent to participate in the study, and the application of the blood concentrate in this study was approved by the responsible Ethics Commission of the state of Hessen, Germany (265/17). The donors were healthy and free of infectious diseases and did not take anticoagulants; nor did they consume alcohol or nicotine. Their ages ranged from 20 to 50 years. In the present study, a blood concentrate of the second generation (i.e., PRF) was used. The blood was prepared in sterile plastic tubes (Plastic S-PRF™ Tubes, Scottsdale, AZ, USA) to obtain a liquid blood concentrate. The authors use the term “PRF” to avoid confusing the readers due to the type of tube used and to help clarify the process. This is because no anticoagulant was prepared in the blood concentrate for this study. The method of preparation used two different relative centrifugal forces (RCFs): (1) a high-RCF PRF (2400 rpm; 8 min; 710 g) and (2) a low-RCF PRF protocol (600 rpm; 8 min; 44 g). After centrifugation, the red blood cell interface was marked (add schematic figure) to separate the PRF into two different phases/regions: the upper plasma clear-yellow phase (upper layer = UL) and the lower red buffy coat phase from the red blood cell interphase of the PRF (buffy coat phase = BC) were transferred separately into sterile 10 mL plastic tubes. The different phases of the low- and high-RCF PRF samples were processed for cellular characterization, the evaluation of growth factor content, and bioactivity analyses (Figure 7). 

### 4.2. Isolation of Human Primary Osteoblasts (pOBs)

Human primary osteoblasts (pOBs) were isolated from excess healthy bone tissue. The application of these cells for this study’s purposes was in accordance with the principle of informed consent of the Ethics Committee of the state of Hessen, Germany. After cutting the bone into small bone fragments with bone scissors and washing the fragments using phosphate-buffered saline (PBS), the bone fragments were transferred to a 6-well plate and cultivated in Dulbecco’s Modified Eagle´s Medium F-12 Ham (DMEM F-12) with 20% fetal calf serum (FCS) and 1% penicillin/streptomycin at 37 °C in a humidified atmosphere (95% oxygen (O_2_) and 5% carbon dioxide (CO_2_)). The medium was changed every other day until osteoblastic cells emerged from the bone and spread on the surfaces of the wells. After a period of 2 to 4 weeks, the cells were detached from the wells of the 6-well plate (0.25%) using trypsin and further cultivated in T-75 cell culture flasks. For the cultivation of the cells passaged in a ratio of 1:2, DMEM F-12 with 10% FCS and 1% penicillin/streptomycin was used. The cells were utilized up to passage 5.

### 4.3. Transwell^TM^ Experiments and Indirect PRF Application

For indirect PRF application, 100.000 human pOBs per well were pre-seeded in 24-well plates and cultivated in DMEM F-12 Ham containing 10% FCS and 1% penicillin/streptomycin for 24 h. Liquid low- and high-RCF PRF matrices were prepared in plastic-coated PRF tubes and each separated into two different phases (PL and BC), as described above. Then, 100 μL of the PL or BC of the low- and high-RCF PRF samples was transferred to Transwell^TM^ inserts with a pore size of 0.4 μm. Wells containing solely human pOBs without the addition of PRF served as controls. After the clotting process of the PRF was completed, 1 mL of DMEM F-12 Ham containing 10% FCS and 1% penicillin/streptomycin was added to the lower compartments of the wells. An additional 100 μL of medium was added to the upper part of the Transwells^TM^. Cells and cell/PRF complexes were cultivated for 2 days. Supernatants were collected after 2 days and stored for an ELISA. Finally, the cells were analyzed for cell viability, and the different PRF phases were fixed for histological and immunohistological staining.

### 4.4. Cell Viability Assay (MTS)

The effect of the different phases of PRF (UL and BC) on the cell viability of the cultured pOBs was examined after 2 days of treatment. After washing the wells with PBS, DMEM F-12 Ham containing 10% FCS, 1% penicillin/streptomycin, and 100 µL of CellTiter 96^®^ AQueous one solution reagent was added. This was followed by an incubation period of 2 h at 37 °C. Then, 100 μL from each well was transferred to a separate 96-well plate and evaluated in a microplate reader according to the manufacturer’s protocol. 

### 4.5. Paraffin Sectioning

After fixing the different coagulated phases of the PRF (UL and BC) in 4% paraformaldehyde, the clots were transferred into embedding cassettes. Subsequently, the PRF clots were processed with a tissue processor and thus prepared for the embedding process. First, a dehydration step with a series of different alcohol concentrations (70%, 96%, 96%, 100%, and 100%) was performed, lasting from 45 min to 1 h each. The clots were then soaked in xylene 3 times for 1 h and embedded in paraffin. After the paraffin embedding process was completed, the samples were sectioned with a rotary microtome and placed in a heating oven for at least 12 h. For each clot, 5 slides with a thickness of 2 μm were prepared. Finally, the slides were stained using various methods and staining procedures. 

### 4.6. Histology and Immunohistochemistry 

One slide of each fixed clot was stained using the hematoxylin–eosin staining method. Therefore, the slides were deparaffinized with a series of xylene and decreasing concentrations of ethanol (100%, 96%, 70%, and 50%). Subsequently, the sections were immersed 3 times in xylene for 5 min each, twice in ethanol (100%), and once in decreasing concentrations of ethanol (96%, 70%, and 50%) for 3 min each. The slides were then submerged in Mayer´s hematoxylin solution to stain the cell nuclei blue. After bluing the samples under running tap water for 10 min, the samples were acidified with eosin for 5 min. The slides were then rinsed under distilled water and placed in a series of increasing concentrations of isopropyl alcohol (70% and 96%) for 2 min each. Thereafter, the slides were soaked twice in isopropyl alcohol (100%) for 3 min. Finally, the samples were covered with Entellan^TM^ to fix the clots with coverslips for a visual analysis. In addition, the different PRF phases (BC and PL) were immunohistochemically stained for neutrophil elastase (ELA) and interferon gamma (INF). Therefore, the samples were deparaffinized using a series of xylene and decreasing concentrations of ethanol (100%, 96%, 70%, and 50%), as previously described. The slides were then treated in a citrate buffer at 96 °C for 20 min. After cooling the slides under running tap water for 10 min, UV-Block was added for 7 min, and two 3 min washing processes in Tris-buffered saline were performed. The specific antibody (ELA, INF), diluted 1:250 (ELA) or 1:100 (INF) in 1% bovine serum albumin (BSA) in PBS, was added for 30 min. After adding polymer horseradish peroxidase (HRP) for 10 min, another washing process was performed. Before placing the slides in water (H_2_O) for 3 min, the slides were covered in 3-amino-9-ethylcarbazole (AEC) for 20 min. Subsequently, the slides were blued with Mayer´s hematoxylin solution for 5 min and washed under running tap water for 10 min. Finally, the slides were covered in Aquatex^®^ to fix the clots with coverslips for a visual analysis. 

### 4.7. Immunofluorescence Staining

To perform immunofluorescence staining on the pOBs treated with different fractions of low- and high-RCF PRF, the cells were fixed in 4% buffered formalin (Roti-Histofix 4%, acid-free pH7, Carl-Roth, Karlsruhe, Germany) and then washed three times with phosphate-buffered saline (PBS). After permeabilizing for intracellular antigens with 0.1% Triton-X100/PBS, the cells were incubated with the specific primary antibody (mouse alpha-smooth muscle actin, Dako) diluted in a 1% BSA/PBS solution (1:2000) for 1 h at room temperature (RT). Fluorescently labeled secondary antibodies (Alexa fluor 488 anti-mouse), diluted in 1% BSA/PBS (1:1000), were used, and the cells were incubated in a dark environment for 1 h at RT. The cell nuclei were counterstained with DAPI diluted in 1% BSA/PBS (1:1000). For documentation, the fluorescent D-LEDI LED illumination system of the Eclipse Ni/E was utilized with the Nikon DS-Ri2 camera (Nikon eclipse Ni/E, Düsseldorf, Germany).

### 4.8. Enzyme-Linked Immunosorbent Assay (ELISA)

Cell culture supernatants were analyzed using ELISA-DuoSets^®^ to quantify the relative protein concentrations of platelet-derived growth factor (PDGF) and transforming growth factor (TGF-β). ELISA DuoSets^®^ were used according to the manufacturer´s instructions. The experiments were performed in duplicate or triplicate per supernatant collected. A microplate reader detected and recorded the optical density of each well. The wavelength was set to 450 nm. Finally, the concentrations were determined, and the results were demonstrated as either absolute values or as a percentage of the control (control = 100%). 

### 4.9. Statistical Analysis

All experiments were conducted using PRF samples from at least three different donors. The presented data are expressed as mean values ± standard deviation values. Statistical significance was determined using either the one-way multifactorial analysis of variance (ANOVA) test or the *t*-test with GraphPad Prism 9 (GraphPad Software Inc.; San Diego, CA, USA). Statistical significance was considered when * *p* < 0.05 and ** *p* < 0.01, and is indicated in the figures.

## 5. Conclusions

This present study characterized the cellular composition and distribution of different layers of blood concentrate matrices generated using two centrifugation protocols and evaluated their effects on the proliferation of primary human osteoblasts. Both the UL and BC layers of PRF demonstrated similar properties in promoting osteoblast proliferation regardless of the centrifugation protocol used. Immunohistochemical analyses revealed differences in cellular composition between the two protocols; however, the proliferative effects on osteoblasts were comparable. This study also highlighted the importance of growth factors in the regenerative process, with low-RCF PRF showing higher levels of TGF-β than high-RCF PRF. These findings contribute to the understanding of the cellular mechanisms underlying the regenerative effects of PRF and provide insights into the optimization of PRF protocols for specific clinical applications. The results suggest that both layers of high- and low-RCF PRF protocols demonstrate similar efficacy in promoting osteoblast proliferation in vitro, making them suitable for bone regeneration procedures. This means that regardless of the type of blood concentrate used, with more or fewer leukocytes in daily practice, the results related to pOB proliferation will be similar. However, further research is needed to validate these findings using more complex in vitro models and experimental studies. Future investigations should explore the potential synergistic effects of combining PRF with other biomaterials to enhance tissue regeneration. Ultimately, this study contributes to ongoing efforts to advance regenerative medicine and improve patient outcomes by providing evidence-based guidance for the optimization of blood concentrate therapies.

## Data Availability

This article does not involve data sharing.
